# 54. Self-Perception of Risk for HIV Acquisition and Calculated Risk for HIV Acquisition Among Active Duty Air Force Members with Newly Diagnosed HIV Infection

**DOI:** 10.1093/ofid/ofab466.054

**Published:** 2021-12-04

**Authors:** Audie B Schmid, Jason Okulicz, Shilpa Hakre, Joseph Yabes, Walter V Mika

**Affiliations:** 1 Brooke Army Medical Center, Fort Sam Houston, TX, San Antonio, Texas; 2 Brooke Army Medical Center, JBSA Fort Sam Houston, TX, San Antonio, Texas; 3 Emerging Infectious Diseases Branch, Walter Reed Army Institute of Research, Silver Spring, MD, Bethesda, Maryland; 4 Brooke Army Medical Center, San Antonio, Texas

## Abstract

**Background:**

Persons may underestimate their risk of HIV infection despite presence of risk factors. Accurate appraisal of HIV risk may assist both patients and providers in preventing HIV acquisition. We evaluated self-perceived risk (SPR) versus calculated risk (CR) of HIV infection in active duty US Air Force (USAF) members with incident HIV infection.

**Methods:**

USAF members with new HIV diagnosis evaluated at a specialty care military medical center between January 2015-March 2020 with available case report forms were included (n=142). Chart reviews were performed and demographic, social, and clinical characteristics were collected from initial Infectious Disease specialty encounters and case report forms. SPR was characterized as Low or High and compared to CR derived by the Denver HIV Risk Score (DHRS) by points based on patient demographic and risk exposure characteristics.

**Results:**

Overall, patients were predominantly male (98%), with a median age of 26 years (IQR 22-30), and the majority (85%) reported same-sex partners (Table 1). Patients more commonly characterized themselves as Low SPR (n=78; 55%) than High SPR (n=64; 45%). Demographic characteristics were similar except a higher proportion of Low SPR patients (29%) were married or partnered compared to High SPR patients (14%; p=0.04). There was no difference in self-reported condom use (≥50% of the time) between Low (63%) and High (72%) SPR patients (p=0.28) and documented history of sexually transmitted infections was similarly high in both groups ( >70%; p=0.85). Previous HIV pre-exposure prophylaxis (PrEP) use was uncommon in both Low SPR (8%) and High SPR (6%) groups. For the evaluation of CR by DHRS (Table 2), both Low and High SPR groups had median scores in the very high risk category (≥50 points) with similar results by test component.

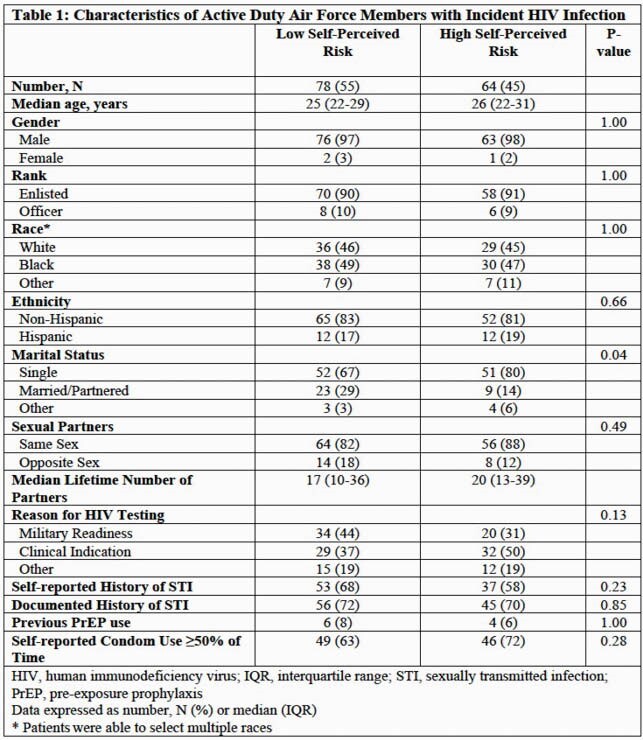

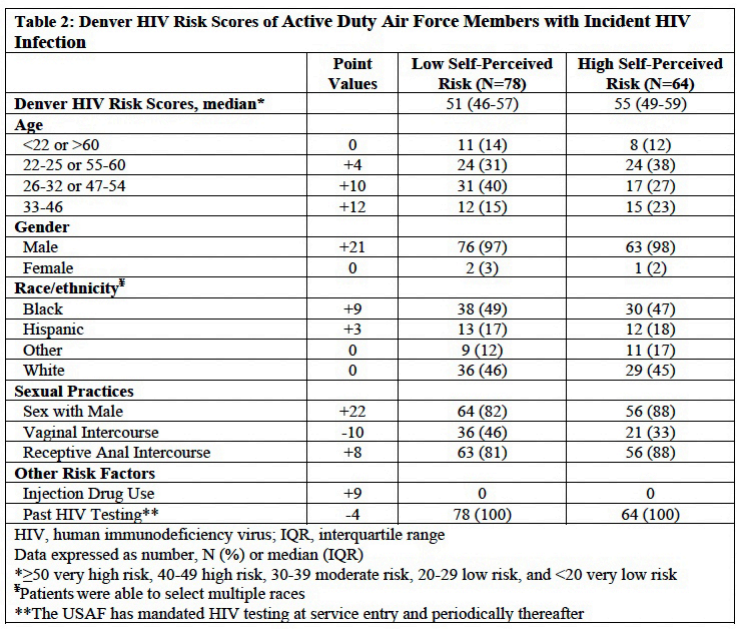

**Conclusion:**

USAF members with incident HIV infection more commonly identified with low SPR despite similar risk behaviors and CRs as high SPR patients. The development of patient education programs and promotion of HIV prevention services including PrEP are needed to reduce incident HIV cases in the USAF. Validated HIV risk calculators like the DHRS may also assist medical providers in identifying candidates for HIV prevention services.

**Disclosures:**

**All Authors**: No reported disclosures

